# Using ALoFT to determine the impact of putative loss-of-function variants in protein-coding genes

**DOI:** 10.1038/s41467-017-00443-5

**Published:** 2017-08-29

**Authors:** Suganthi Balasubramanian, Yao Fu, Mayur Pawashe, Patrick McGillivray, Mike Jin, Jeremy Liu, Konrad J. Karczewski, Daniel G. MacArthur, Mark Gerstein

**Affiliations:** 1 Program in Computational Biology and Bioinformatics, Yale University, New Haven, CT 06520 USA; 2 Molecular Biophysics and Biochemistry Department, Yale University, New Haven, CT 06520 USA; 3 Analytic and Translational Genetics Unit, Massachusetts General Hospital, Boston, MA 02114 USA; 4 Program in Medical and Population Genetics, Broad Institute of MIT and Harvard, Cambridge, Massachusetts 02142 USA; 5 Department of Computer Science, Yale University, New Haven, CT 06520 USA; 6 Regeneron Genetics Center, Tarrytown, NY 10591 USA; 7 Bina Technologies, Part of Roche Sequencing, Belmont, CA 94002 USA

## Abstract

Variants predicted to result in the loss of function of human genes have attracted interest because of their clinical impact and surprising prevalence in healthy individuals. Here, we present ALoFT (annotation of loss-of-function transcripts), a method to annotate and predict the disease-causing potential of loss-of-function variants. Using data from Mendelian disease-gene discovery projects, we show that ALoFT can distinguish between loss-of-function variants that are deleterious as heterozygotes and those causing disease only in the homozygous state. Investigation of variants discovered in healthy populations suggests that each individual carries at least two heterozygous premature stop alleles that could potentially lead to disease if present as homozygotes. When applied to de novo putative loss-of-function variants in autism-affected families, ALoFT distinguishes between deleterious variants in patients and benign variants in unaffected siblings. Finally, analysis of somatic variants in >6500 cancer exomes shows that putative loss-of-function variants predicted to be deleterious by ALoFT are enriched in known driver genes.

## Abstract (web summary)

Variants causing loss of function (LoF) of human genes have clinical implications. Here, the authors present a method to predict disease-causing potential of LoF variants, ALoFT (annotation of Loss-of-Function Transcripts) and show its application to interpreting LoF variants in different contexts.

## Introduction

One of the most notable findings from personal genomics studies is that all individuals harbor loss-of-function (LoF) variants in some of their genes^1^. A systematic study of LoF variants from the 1000 Genomes Project revealed that there are over 100 putative LoF (pLoF) variants in each individual^2–4^. Recently, a larger study aimed at elucidating rare LoF events in 2636 Icelanders generated a catalog of 1171 genes that contain either homozygous or compound heterozygous LoF variants with a minor allele frequency less than 2%^5^. Thus, several genes are knocked out either completely or in an isoform-specific manner. The discovery of protective LoF variants associated with beneficial traits and their potential to enable identification of valuable drug targets has fueled an increased interest in pLoF variants. For example, nonsense variants in *PCSK9* are associated with low low-density lipoprotein﻿ (LDL) levels^6^, which prompted the active pursuit of the inhibition of *PCSK9* as a potential therapeutic for hypercholesterolemia^7, 8^ and led to the development of two drugs that have been recently approved by the FDA. Other examples include nonsense and splice mutations in *APOC3* associated with low levels of circulating triglycerides, a nonsense mutation in *SLC30A8* resulting in about 65% reduction in risk for Type II diabetes, two splice variants in the Finnish population in *LPA* that protect against coronary artery disease, and two LoF-producing splice variants and a nonsense mutation in *HAL* associated with increased blood histidine levels and reduced risk of coronary artery disease^9–12^.

About 12% of known disease-causing mutations in the Human Gene Mutation Database (HGMD) are due to nonsense mutations^13^. Even though premature stop variants often lead to loss of function and are thus deleterious, predicting the functional impact of premature stop codons is not straightforward. Aberrant transcripts containing premature stop codons are typically removed by nonsense-mediated decay (NMD), an mRNA surveillance mechanism^14^. However, a recent large-scale expression analysis demonstrated that 68% of predicted NMD events due to premature stop variants are unsupported by RNA-Seq analyses^15^. Moreover, premature stop codons in the last exon are generally not subject to NMD. A study aimed at understanding disease mutations using a 3D structure-based interaction network suggests that truncating mutations can give rise to functional protein products^16^. Furthermore, when a variant affects only some isoforms of a gene, it is difficult to infer its impact on gene function without the knowledge of the isoforms that are expressed in the tissue of interest and how their levels of expression affect gene function. Finally, loss of function of a gene might not have any impact on the fitness of the organism.

While there are several algorithms to predict the effect of missense coding variants on protein function, there is a paucity of methods that are applicable to nonsense variants^17–19^. Additionally, current prediction methods that infer the pathogenicity of variants do not take into account the zygosity of the variant^20, 21^. The majority of pLoF variants in healthy cohorts are heterozygous. It is likely that a subset of these variants will cause disease as homozygotes.

Here we present a pipeline called ALoFT (Annotation of Loss-of-Function Transcripts), that provides extensive annotation of pLoF variants. Furthermore, we developed a prediction model to classify pLoF variants into three classes: those that are benign, those that lead to recessive disease (disease-causing only when homozygous) and those that lead to dominant disease (disease-causing as heterozygotes). Finally, we validated the prediction model by applying ALoFT to known disease mutations in Mendelian diseases, autism, and cancer.

## Results

### ALoFT pipeline

We have developed a pipeline called ALoFT to annotate pLoF variants. In this study, we included premature stop-causing single-nucleotide polymorphisms (SNPs), frameshift-causing indels and variants affecting canonical splice sites as pLoF variants, also referred to as protein truncating variants. An overview of the pipeline is shown in Supplementary Fig. 1. The main features of ALoFT include (1) functional domain annotations; (2) evolutionary conservation; and (3) biological networks. For comprehensive functional annotation, we integrated several annotation resources such as PFAM and SMART functional domains^22, 23^, signal peptide and transmembrane annotations, post-translational modification sites, NMD prediction^24, 25^, and structure-based features such as SCOP domains and disordered residues. For evolutionary conservation, ALoFT outputs variant position-specific GERP scores, which is a measure of evolutionary conservation^26^ and d*N*/d*S* values (ratio of missense to synonymous substitution rates) for macaque and mouse that are computed from human-macaque and human-mouse orthologous alignments, respectively. In addition, we evaluated if the region removed due to the truncation of the coding sequence is evolutionarily conserved based on constrained elements^27^. ALoFT includes network features shown to be important in disease prediction algorithms: a proximity parameter that gives the number of disease genes connected to a gene in a protein–protein interaction network and the shortest path to the nearest disease gene^2, 28^. The pipeline also includes features to help identify erroneous LoF calls, potential mismapping, and annotation errors, because LoF variant calls have been shown to be enriched for annotation and sequencing artifacts^2^. A description of all the annotations provided by ALoFT is included in Supplementary Table 1 (details in Methods). Documentation and link to source code can be found at aloft.gersteinlab.org.

**Fig. 1:**
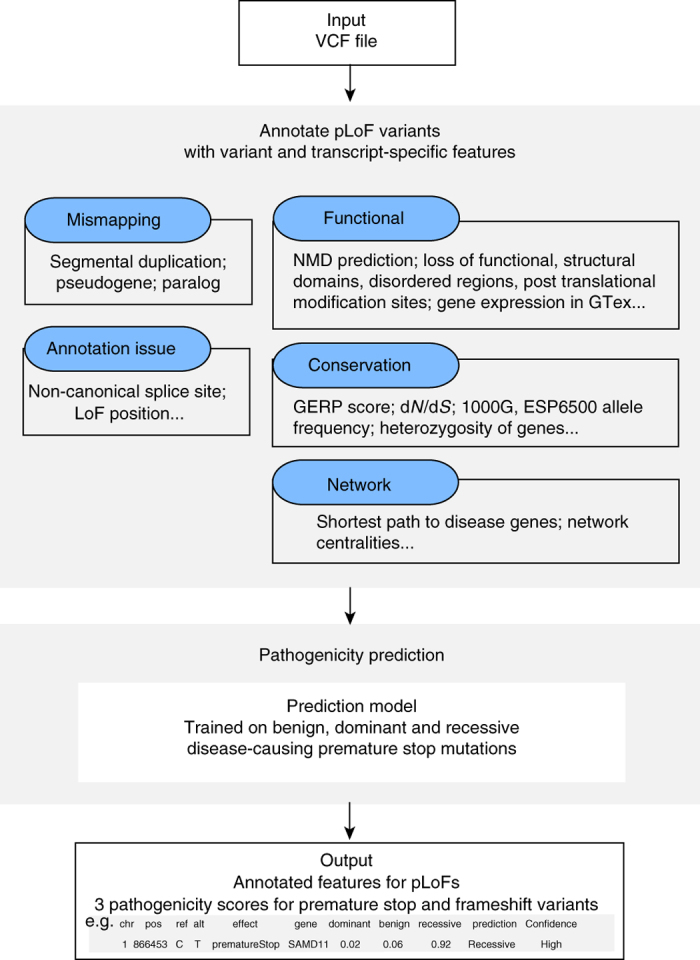
Schematic workflow. ALoFT uses a VCF file as input and annotates premature stop, frameshift-causing indel and canonical splice-site mutations with functional, conservation, and network features. ALoFT also flags potential mismapping and annotation errors. Using the annotation features, ALoFT predicts the pathogenicity (as either benign, recessive, or dominant disease-causing) of premature stop and frameshift mutations based on a model trained on known data. ALoFT can also take as input a five-column tab-delimited file containing chromosome, position, variant ID, reference allele, and alternate allele as its columns

Using the annotations output by ALoFT as predictive features (Fig. 1, Supplementary Data 1), we developed a prediction method to infer the pathogenicity of pLoF variants. To build the ALoFT classifier, we used three classes of premature stop variants as training data: benign variants, dominant disease-causing variants, and recessive disease-causing variants (Supplementary Table 2). The benign set includes homozygous premature stop variants discovered in a cohort of 1092 healthy people, Phase1 1000 Genomes data (1KG). Homozygous premature stop mutations from HGMD that lead to recessive disease and heterozygous premature stop variants in haplo-insufficient genes that lead to dominant disease represent the two disease classes^3, 28^. In addition to loss-of-function effects, truncating mutations can also lead to gain of function. However, gain-of-function mutations are difficult to model systematically as the effect of a variant can only be understood in the context of the biology of the gene and can vary widely for different genes and gene classes. In order to minimize errors that might arise due to inadequate modeling of gain-of-function effects and to focus on LoF, we only use predicted haploinsufficient genes as the training data for the dominant model. We built the ALoFT classifier to distinguish among the three classes using a random forest algorithm^29^ (details in Methods). For each mutation, ALoFT provides three class probability estimates, and we obtain good discrimination between each class. The prediction output provides the three scores for each pLoF variant that correspond to the probability of the pLoF being benign, dominant or recessive disease-causing allele. In addition, ALoFT also provides the predicted pathogenicity. The pathogenic effect of pLoF variant is assigned to the class that corresponds to the maximum score.

### Evaluation of the classifier

The average multiclass test area under the curve (AUC) with 10-fold cross-validation is 0.97. The precision for the three classes are as follows: dominant = 0.86, recessive = 0.86, and benign = 0.96. The recall for the three classes are as follows: dominant: 0.71; recessive: 0.95; and benign: 0.96.

The classifier is robust to the choice of training data sets (Supplementary Table 3, details in Methods). Though trained with premature stop SNVs, our method is also applicable to frameshift indels. We applied ALoFT to classify pathogenic indels in HGMD. 99.4% of HGMD disease-causing frameshift indels are predicted to be pathogenic based on the maximum ALoFT score.

We analyzed the importance of the various features to the classification (Supplementary Fig. 2). The global allele frequency of variants in the Exome Aggregation Consortium, ExAC, a data set comprising sequence variations obtained from an analysis of 60,706 unrelated individuals of diverse ethnicities (ExAC^30^, http://exac.broadinstitute.org), appears to be the most important feature for classification. When we removed this feature and other features related to allele frequency (i.e., features related to variants in both ExAC and Exome Sequencing Project, ESP) and retrained the random forest model, the classifier still performs well with an average multiclass test AUC of 0.93. (The precisions for the three classes are as follows: dominant = 0.84, recessive = 0.80, and benign = 0.75). We also systematically evaluated the classifier using models trained on varying sets of features (Supplementary Table 4). Overall, we find that the classification is not driven by any single feature and integrating many features improves prediction accuracy.

### Validation of the classifier

We applied ALoFT to elucidate the pathogenicity of pLoF variants in various disease scenarios. Using case studies, we show that ALoFT provides robust predictions for the effect of pLoFs.

### Understanding pLoFs in Mendelian disease

**Fig. 2:**
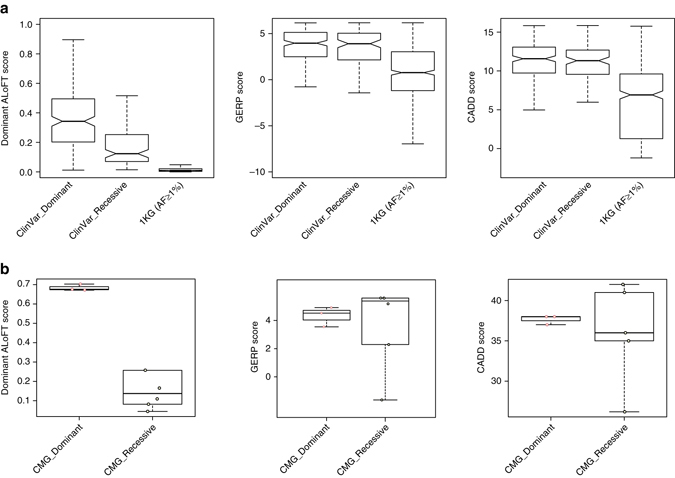
ALoFT classification of pathogenic premature stop variants from Mendelian disease studies. **a** Dominant ALoFT, GERP, and CADD scores for ClinVar and 1KG common (AF ⩾ 1%) variants. All training variants are excluded. Average benign ALoFT scores are 0.097 and 0.115, respectively, for ClinVar dominant and recessive data sets. AF denotes Allele Frequency. 1KG stands for 1000 Genomes Phase1 data. **b** Dominant ALoFT, GERP, and CADD scores for pathogenic variants from the CMG studies. In these plots, the center line represents the median value of the data, the box goes from the first quartile to the third quartile. The lower whisker goes from Q1 to the smallest non-outlier in the data set, and the upper whisker goes from Q3 to the largest non-outlier in the data set. In addition, the data points are also plotted as *open circles*

We evaluated ALoFT by predicting the effect of known disease-causing premature stop mutations from ClinVar^31^ (details in Methods) and predicted the mode of inheritance and pathogenicity of all of truncating variants (Fig. 2a). ALoFT is clearly able to distinguish between pLoFs that lead to disease in a heterozygous state vs. those that do so only in a homozygous state. Our method shows that heterozygous disease-causing variants have significantly higher dominant disease-causing scores than the homozygous disease-causing variants (*p*-value: 1.3e-13; Wilcoxon rank-sum test). We used two other measures, GERP score, which is a measure of evolutionary conservation, and CADD score, which gives a measure of pathogenicity, to classify recessive vs. dominant pLoF variants^32^. Both CADD (*p*-value: 0.13; Wilcoxon rank-sum test) and GERP (*p*-value: 0.49; Wilcoxon rank-sum test) scores are not able to discriminate between recessive and dominant disease-causing mutations (Fig. 2a). We also tested our method on a smaller data set from the Center For Mendelian Genomics studies^33^ and were able to correctly recapitulate the pathogenic effect of pLoF variants and their inheritance pattern (Fig. 2b).

### Understanding de novo pLoFs implicated in autism

**Fig. 3:**
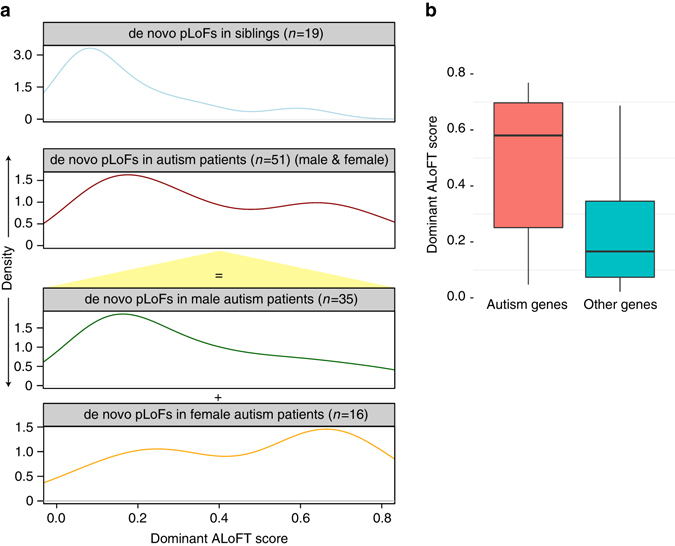
ALoFT classification of de novo premature stop variants from autism studies. **a** The top two panels show the ALoFT dominant scores of de novo premature stop mutations in autism patients and siblings; mutations in patients are further separated by gender, as shown in the bottom two panels. **b** ALoFT dominant prediction scores for autism de novo pLoFs in confident risk genes. In this plot, the *center line* represents the median value of the data, the box goes from the first quartile to the third quartile. The lower whisker goes from Q1 to the smallest non-outlier in the data set, and the upper whisker goes from Q3 to the largest non-outlier in the data set

De novo pLoF SNPs have been implicated in autism based on analysis of sporadic or simplex families (families with no prior history of autism)^34–37^. We applied our method to de novo pLoF mutations discovered in these studies. Each individual carries about one de novo premature stop variant (Supplementary Table 5). Our method shows that the proportion of dominant disease-causing de novo LoF events is significantly higher in autism patients vs. siblings of patients with autism (Fig. 3a; *p*-value: 8.4e−4; Wilcoxon rank-sum test).

Autism spectrum disorder is known to be four times more prevalent in males than in females suggesting a protective effect in females. Previous studies show that a higher mutational burden of non-synonymous mutations is ascertained in females with autism spectrum disorder^38^. Therefore, we investigated differences in the impact of de novo pLoF variants in male vs. female autism patients. We observed a similar pattern for pLoF mutations as has been found for missense variants—female probands have a higher proportion of predicted deleterious de novo pLoF variants than male probands (Fig. 3a; *p*-value: 0.03; Wilcoxon rank-sum test). Supplementary Data 2 includes the ALoFT predictions for de novo pLoF variants. A recent study based on exome sequencing of 3871 autism cases delineated 33 risk genes at FDR < 0.1^39^. We observed that de novo pLoF mutations in the 33 risk genes of the autism patients have higher dominant disease-causing scores than the de novo pLoF variants in other genes (Fig. 3b; *p*-value: 5e−3; Wilcoxon rank-sum test). Thus, ALoFT predictions corroborate the role of de novo pLoF variants in autism as shown by others using entirely different approaches.

### Identification of pathogenic somatic LoF variants in cancer

**Fig. 4:**
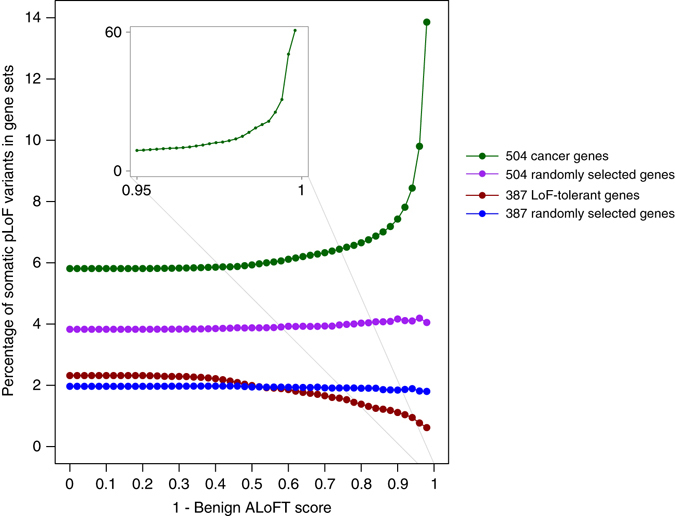
ALoFT classification of somatic premature stop variants. The fraction of mutations occurring in various gene categories (*Y* axis) as a function of predicted diseasing-causing score for cancer somatic premature stop variants (*X* axis). Disease-causing score is calculated as (1—predicted benign ALoFT score). We calculated the fraction of somatic premature stop mutations in 504 known cancer driver genes and 504 randomly selected genes. To ensure that the cancer driver genes and the randomly selected genes have similar length distributions, the 504 random genes were selected from genes with matched length. Similarly, we compared the fraction of somatic premature stop mutations in 397 LoF-tolerant genes and 397 randomly selected genes with similar length distribution. LoF-tolerant genes are genes that have at least one homozygous LoF variant in at least one individual in the 1KG cohort

We applied our prediction method to infer the effect of somatic premature stop variants (somatic pLoFs) from a compilation of 6535 cancer exomes^40^. As shown in Fig. 4, somatic pLoFs are enriched in known cancer driver genes compared to randomly sampled genes of matched lengths. Moreover, deleterious somatic LoFs are strongly enriched in driver genes and depleted in LoF-tolerant genes (genes that contain at least one homozygous LoF variant in the 1KG population). In the context of somatic mutations, variant zygosity, or distinguishing between ‘dominant’ and ‘recessive’ disease-causing mutations, is not always relevant. Cancer cells may show aneuploidy and cellular heterogeneity. Therefore, for the evaluation of somatic mutations, we define an overall measure of deleteriousness as (1-benign ALoFT score) on the *X* axis of Fig. 4.

**Fig. 5:**
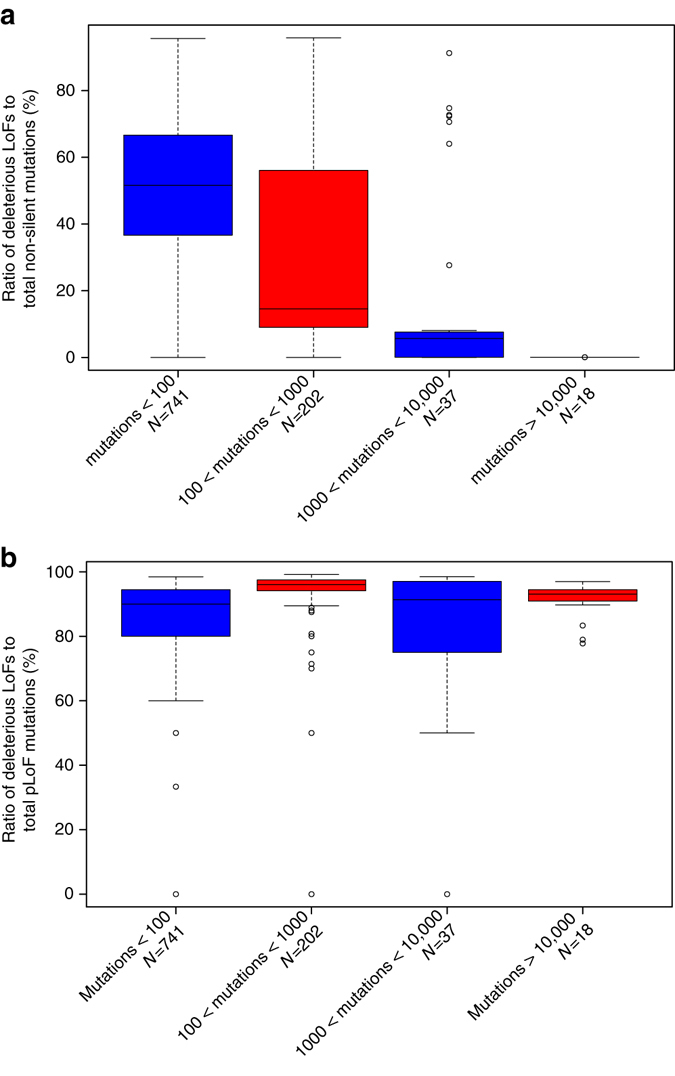
Accumulation of deleterious LoF variants. **a** The *top panel* depicts the accumulation of deleterious LoF variants vs. total non-silent variants. For this analysis, following four different intervals were defined based on mutation burden: <100 mutations (*N* = 741 samples), 100–1000 mutations (*N* = 202 samples), 1000–10,000 mutations (*N* = 37 samples), and >10,000 mutations (*N* = 18). Non-silent variants include missense variants and putative loss-of-function (pLoF) variants. **b** The *bottom panel* depicts the accumulation of deleterious LoF variants vs. total pLoF variants. In both *box plots*, the *center line* represents the median value of the data, the box goes from the first quartile to the third quartile. The lower whisker goes from Q1 to the smallest non-outlier in the data set, and the upper whisker goes from Q3 to the largest non-outlier in the data set. Outliers are indicated by the *plotted points*

We also evaluated ALoFT as a tool for distinguishing driver LoF mutations from passenger LoF mutations in tumors with high mutation burden. We observed a decrease in deleterious LoF mutations with increasing total mutational burden (Fig. 5a). However, the ratio of deleterious LoFs to total pLoFs displayed no significant trend across groups (Fig. 5b). The ratio of deleterious LoF mutations to total pLoF mutations is consistently high across groups (84%). This may indicate that driver LoF events tend to arise early in tumor development.

**Fig. 6:**
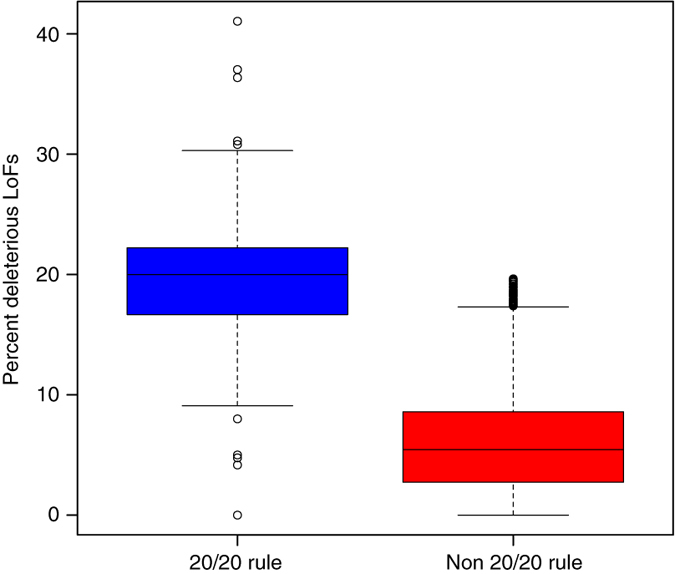
Proportion of deleterious LoFs in tumor suppressor genes. Tumor suppressor genes were identified using Vogelstein’s 20/20 rule. Samples containing at least 20 somatic mutations were included in this analysis. In the *box plots*, the *center line* represents the median value of the data, the box goes from the first quartile to the third quartile. The lower whisker goes from Q1 to the smallest non-outlier in the data set, and the upper whisker goes from Q3 to the largest non-outlier in the data set. Outliers are indicated by the *plotted points*

To classify genes as tumor suppressors, Vogelstein et al.^41^ proposed a “20/20” rule, whereby a gene is classified as a tumor suppressor if >20% of the observed mutations in that gene are inactivating mutations. Among the 210 genes that met 20/20 rule criteria, 87% of pLoF mutations affecting these genes were deleterious LoFs, representing 21% of total mutations. By comparison, only 6% of mutations were deleterious LoFs among 11,892 genes that did not meet 20/20 criteria (*p* < 0.001, chi-squared test) (Fig. 6). A list of these genes is provided as Supplementary Data 3. In cases where genes display a high somatic pLoF rate but low somatic deleterious LoF rate, ALoFT may be used to identify potential false-positive driver genes predicted by the 20/20 rule.

### Distinguishing between benign and pathogenic pLoFs

**Fig. 7:**
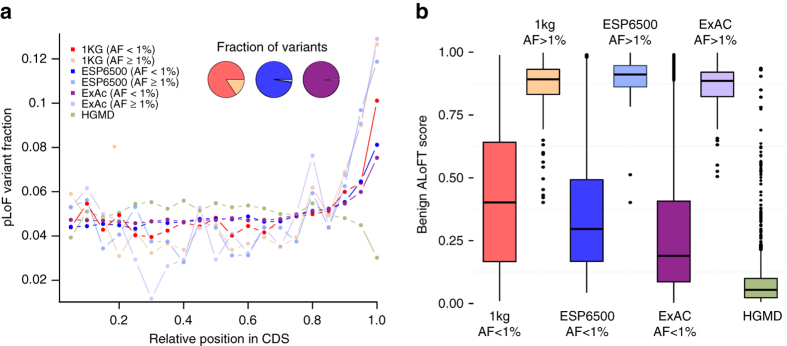
pLoFs in last exons. **a** Position of premature stop variants in coding transcripts. Compared to HGMD variants, both common and rare 1KG, ESP6500, and ExAC variants are enriched in the last 5% of the coding sequence. AF, allele frequency, pLoF, putative loss-of-function variant, and CDS, coding sequence. Variants at allele frequency <1% are considered to be rare variants. Variants with at least 1% allele frequency are considered common. **b** Predicted benign scores for premature stop variants in the last coding exons. In the *box plots*, the *center line* represents the median value of the data, the box goes from the first quartile to the third quartile. The lower whisker goes from Q1 to the smallest non-outlier in the data set, and the upper whisker goes from Q3 to the largest non-outlier in the data set. Outliers are indicated by the *plotted points*. Training variants are excluded in this plot

Finally, we applied ALoFT to predict the effect of premature stop variants in the final exons of protein-coding genes. It is often assumed that premature stop variants in the last coding exon are likely to be benign because they could escape NMD; as a result, in many cases, the effect will be the expression of a truncated protein rather than a complete loss of function. However, several examples of disease-causing mutations in the last exon are known^42^. Therefore, we applied ALoFT to see if we could distinguish between benign and disease-causing LoF variants in the last coding exon. To this end, we applied ALoFT to understand the effect of pLoF variants in ESP6500, ExAC, and HGMD data sets. A higher proportion of rare variants is observed in ESP6500 and ExAC cohorts due to their larger sample size and higher sequencing depth (Fig. 7a). A large number of both common and rare premature stop variants are seen at the end of the coding genes in the 1KG, ESP6500, and ExAC data sets. In contrast, fewer disease-causing HGMD variants are seen at the ends of coding genes (Fig. 7b). ALoFT predicts that both common and rare premature stop variants in the last coding exon in the 1KG, ESP6500, and ExAC cohort are likely to be benign, whereas HGMD mutations in the last coding exon tend to be disease-causing (Fig. 7b). Thus, ALoFT is able to differentiate between rare benign premature stop variants seen in healthy individuals and rare disease-causing HGMD alleles.

### pLoFs in an individual genome

**Fig. 8:**
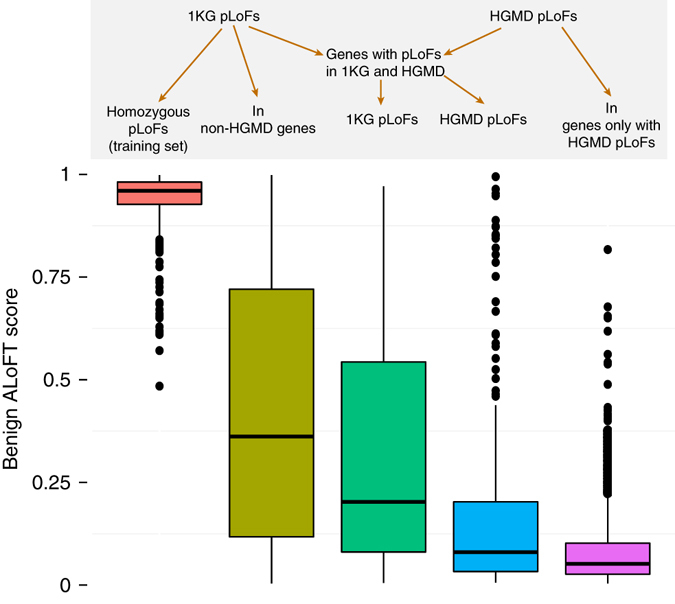
ALoFT classification of 1000 Genomes and HGMD variants. Benign scores for premature stop variants in 1KG and HGMD. For this plot, we randomly selected one variant per gene. The “Benign pLoFs” set includes homozygous premature stop variants discovered in 1KG. The third (*dark green*) *box plot* pertains to premature stop variants in healthy 1KG individuals occurring in disease-causing genes obtained from HGMD. The fourth (*blue*) *box plot* pertains to pLoF variants in the subset of HGMD genes where 1KG pLoFs are also seen. “1KG pLoFs in non-HGMD genes” include 1KG variants not in HGMD genes, i.e., non-disease genes. “In genes only with HGMD pLoFs” includes HGMD variants in only those disease genes where 1KG pLoFs are not seen. In the *box plots*, the *center line* represents the median value of the data, the box goes from the first quartile to the third quartile. The lower whisker goes from Q1 to the smallest non-outlier in the data set, and the upper whisker goes from Q3 to the largest non-outlier in the data set. Outliers are indicated by the *plotted points*

The above case studies clearly illustrate the validity of the ALoFT score in elucidating the effect of pLoF variants. In order to estimate the number of pLoF disease alleles in a healthy individual, we applied ALoFT to premature stop variants from the 1KG and ExAC data sets. The predicted benign score for pLoFs in 1KG has a wide range of values (Fig. 8, Supplementary Data 4). Furthermore, due to differences in sequencing coverage and variant calling approaches, the number of potential disease pLoFs per individual varies among datasets. In general, the number increases with higher coverage and larger cohorts where joint variant calling methods result in improved sensitivity in the identification of rare variants. To conservatively estimate a lower bound for per individual statistics (Methods), we applied a stringent filtering strategy to restrict to high confidence pLoFs. On average, each individual is a carrier of at least two rare heterozygous premature stop alleles that are predicted to be disease-causing in the homozygous state (Supplementary Table 6) based on the 1KG Phase1 data. Current estimates of the genetic burden of disease alleles (all types of variation, including LoFs) in an individual vary widely, ranging from 1.1 recessive alleles per individual to 31 deleterious alleles^43–47^. In connection with this, it should be noted that the referenced studies are based on diverse methods of identifying variants ranging from targeted panel-based candidate gene studies to whole-genome sequencing. The estimation of the number of deleterious pLoF alleles can be affected by a number of confounding factors that include incomplete penetrance of disease alleles, variable expressivity, compensatory mutations, marginal variant calls, and imperfect training data sets (Methods).

Next, we looked at premature stop variants in the 1KG cohort in known disease-causing genes. We find that variants in 1KG are more likely to be benign compared to known disease-causing mutations in the same genes (Fig. 8; green vs. blue boxes, *p*-value: 6.9e−9). Our results provide a possible rationale for this observation. Firstly, variants predicted to be benign in 1KG often affect isoforms that are different from the isoforms containing the disease-causing HGMD variant. This suggests that LoFs in healthy individuals may affect minor isoforms (Supplementary Fig. 3). About 12.4% of premature stop variants in the presumed healthy 1KG individuals occur in known disease genes and the disease-causing variants in the same genes are on different isoforms. Secondly, some variants predicted to be benign in 1KG occur in the last exon or later in the protein-coding transcript relative to the disease-causing variant in the same transcript. The effect of such variants is possibly the production of truncated proteins that are sufficiently functional. Lastly, a majority of 1KG variants seen in disease genes are predicted to be disease-causing only if they are homozygous. However, they occur as rare heterozygous variants in the 1KG cohort.

Mutations in HGMD are assumed to be disease-causing. However, some mutations are predicted to be benign by ALoFT (Fig. 8). It is known that disease databases include incorrect disease annotations and common variants and about 27% of variants were excluded by Bell et al.^43^ in their estimate of carrier burden for severe recessive diseases. However, overall only 0.67% of HGMD premature stop mutations are predicted to be benign. Supplementary Fig. 4 shows that most mutations predicted to be benign by ALoFT are seen at higher allele frequencies than those predicted to be in the dominant and recessive classes. Of the 119 pLoF autosomal variants in HGMD predicted to be benign by ALoFT, 32 variants are in Filaggrin, *FLG*. *FLG* LoF mutations are linked to susceptibility to atopic dermatitis, a skin condition leading to eczema. Eczema is a complex trait and the resulting phenotypes are highly variable due to the interplay of environmental and genetic factors. A recent study showed that individuals with bi-alleleic null variants of *FLG* do not always have atopic dermatitis^48^.

A study on British Pakistanis with related parents identified 781 genes containing rare homozygous LoF variants^49^. They found homozygous LoF variants in recessive Mendelian disease genes; however, carriers of most of these homozygous LoF variants do not have the disease phenotype. We applied ALoFT to classify these homozygous LoF variants. Of the 22 variants for which ALoFT provides predictions, 3 are predicted to be benign and none of them were predicted to lead to disease by the dominant mode of inheritance. However, 19 homozygous variants are indeed predicted to lead to disease with a recessive mode of inheritance (Supplementary Data 5). The lack of a discernible phenotype could be due to incomplete penetrance of the mutations or due to modifier effects. The penetrance of some disease mutations are also known to be age dependent and sex dependent^50^. While studies in consanguineous populations have been used to identify recessive disease genes^51^, the absence of disease provides an opportunity to look for modifiers in their genetic background.

## Discussion

In summary, we describe ALoFT, a tool for predicting the impact of pLoF variants. In the context of a diploid model, it may be used to determine whether pLoF variants are likely to lead to recessive or dominant disease. Better identification and characterization of pLoF variants have both diagnostic and therapeutic implications. ALoFT allows for the identification and prioritization of high-impact putative disease-causing pLoF variants in individual genomes. Integrating benign LoF variants with phenotypic information will help us to identify protective variants that are valuable drug targets^52^. Gene functions important for species propagation might actually be deleterious as one ages; thus, LoF variants in such genes provide an intriguing avenue to discover targets for aging-related diseases^53^. Lastly, diseases caused by LoF variants provide opportunities for targeted therapy using drugs that either enable read-through of the premature stop, thus restoring the function of the mutant protein, or NMD inhibitors that prevent degradation of the LoF-containing transcript by NMD^54, 55^. This is especially useful in the context of rare diseases where targeting the same molecular phenotype leading to different diseases alleviates the need to design a new drug for each individual disease. Further work will be needed both to correlate the predictions of ALoFT with experimental assays of protein LoF and to study the phenotypic impact of heterozygous and homozygous LoF variants in large clinical cohorts.

## Methods

### Overview of ALoFT annotation pipeline

ALoFT provides extensive annotation for SNPs that introduce a premature stop codon, SNPs affecting splice sites, and indels that lead to frameshift. Initial sequence-based annotation of coding variants is performed by the Variant Annotation Tool^56^ (VAT). The output of VAT is augmented with various features specific to pLoF variants. The input files can be in VCF format or a tab-delimited 5-column file that includes chromosome, variant position, variant ID, reference allele, and alternate allele. LoF variants annotated with various features are output as three separate files: a VCF-formatted file containing summarized annotations, a Tab-delimited file containing extensive annotations for premature stop variants and indels leading to frameshift, a tab-delimited file containing annotations for variants that affect the canonical splice sites.

The output of ALoFT annotation pipeline is discussed below and the overview of the pipeline is shown in Supplementary Fig. 1.

### Feature annotation

In total, we used 108 features to train our model (Supplementary Data 1). In terms of functional features, we annotated domains affected by pLoF variants with PFAM and SMART domain information. The 3D structure of a protein is essential for proper folding and function of proteins. Therefore, we incorporated two structure-based features, SCOP domains, and disordered residues, into our pipeline. In addition, we annotated signal peptide and trans-membrane domains. PFAM, SCOP, signal peptide, and trans-membrane domain annotations were obtained by querying Ensembl Release 73 using the Ensembl PERL API^57^. Post-translationally modified residues (phosphorylated, acetylated, and ubiquitinated sites) are annotated based on data from PhosphositePlus^25^. Disordered residues are known to be important in protein–protein interaction surfaces and have been implicated in disease-causing mechanisms^58, 59^. We obtained disordered residues in proteins using DISOPRED^24^. For all functional features, we addressed the following questions: (1) does the premature stop variant affect a functional feature? and (2) are functional, structural, or other domains removed due to truncation?

Nonsense-mediated decay (NMD) is a cellular surveillance mechanism whereby transcripts containing premature stop codons are removed to prevent aberrant transcripts and protein products. NMD can be used as a feature to assess whether a transcript containing a pLoF variant will be functional. We therefore included NMD prediction as a functional feature and identified transcripts containing pLoF variants as candidates for NMD if the distance of the premature stop from the last exon–exon junction was >50 base pairs.

As network features, we calculated proximity parameters for each pLoF-affected gene that correspond to the number of disease genes directly connected to it in a protein–protein interaction network. Human protein–protein interaction networks were downloaded from BioGrid^60^ (the version used is BIOGRID-ORGANISM-Homo_sapiens-3.2.95). Dominant and recessive disease genes were obtained from lists curated from OMIM^61–63^. Shortest path from a gene to the nearest disease gene in the protein–protein interaction network is also included in the ALoFT output.

The evolutionary features considered by ALoFT include the GERP score of the pLoF variant position. In the case of indels, the mean GERP score is provided. In addition, ALoFT evaluates the evolutionary conservation of the region that is lost due to the truncation. This is calculated as the percentage of coding region lost that occurs in GERP-constrained elements. d*N*/d*S* values for human-macaque and human-mouse orthologs were obtained from Ensembl using Biomart.

ALoFT also includes all annotation features derived from VAT. This includes transcript-specific annotation of the coding SNP. In addition, ALoFT provides allele frequency information for the variants based on reference population studies. Specifically, ALoFT output includes allele frequency information for LoF variants from the Phase1 of 1000 Genomes Project (1KG), ESP6500, as well as ExAC data sets. 1KG includes genetic variation data obtained from whole genome and exome sequencing of 1092 healthy individuals. ESP6500 consists of genetic variants from exome sequencing of a cohort of 2203 African-American and 4300 European-American unrelated individuals enrolled in the National Heart, Lung, and Blood Institute Exome Sequencing Project^3^. The ESP6500 data set was downloaded from Exome Variant Server, NHLBI GO Exome Sequencing Project (ESP), Seattle, WA (URL: http://evs.gs.washington.edu/EVS/) (8 November 2013). Version 0.3 of the ExAC data set was downloaded from http://exac.broadinstitute.org/, containing 60,706 unrelated individuals sequenced as part of various disease-specific and population genetic studies^30^. An overview of all the features output by ALoFT is shown in Supplementary Table 1.

To account for gene conservation, we calculated synonymous and non-synonymous SNP density based on variation data from 1KG, average GERP scores of synonymous and non-synonymous SNPs, the percentage of synonymous and non-synonymous SNPs in GERP-constrained elements, the percentage of coding transcript overlapping with constrained GERP elements, and average heterozygosity for synonymous and non-synonymous SNPs in 1KG. Gene centrality scores were obtained for various networks from Khurana et al.^64^ Transcript expression levels in 25 tissues from GTex^65^. For each transcript, we calculated the average expression values across individuals for a particular tissue. Tissue specificity is calculated using a Shannon entropy-based method^66^. Number of validated miRNA binding sites per gene were obtained from miRWalk^67^. Average heterozygosity was calculated as $\frac{{{\sum} {2pq} }}{l}$, where *p* is minor allele frequency, *q* is the reference allele frequency, *l* is the length of the coding transcript.

### Accounting for annotation errors and mismapping errors

In order to reduce mismapping errors, ALoFT flags potential false-positive variant calls by identifying homologous regions in the genome where the potential for mismapping is high. This includes variants in segmentally duplicated regions, variants in genes that have paralogs, and variants in genes that have pseudogenes. 51,599 regions of the human genome are annotated as segmentally duplicated regions that are at least 1 kb in length and whose sequences are >90% identical. Paralogs of human genes were obtained from Ensembl, with 11,658 genes having paralogs. Pseudogene information was derived from the GENCODE pseudogene resource^68^. 3392 genes have pseudogenes.

Variants that lead to a premature Stop codon, indels that lead to frameshift and variants in splice sites are annotated as pLoF variants based on sequence annotation and are assumed to lead to loss of function. However, this assumption is not always valid. Categories of LoF annotation errors have been evaluated and elucidated in the first systematic catalog of loss-of-function genes^2^. Thus, the various ways that an inferred LoF annotation may be incorrect are captured by ALoFT using several flags. *lof_anc*: indicates that the pLoF variant allele is the same as the ancestral allele. Evolutionarily conserved alleles imply that they are likely to be biologically important and thus represent functional alleles. Therefore, when the pLoF variant is same as the ancestral allele, we believe that it is a functional allele. *near_start*: The variant is in the first 5% of the coding sequence. *near_end*: The variant is in the last 5% of the coding sequence. *alt_canonical_site*: SNPs in splice sites are flagged as potentially not LoF when the alternate allele represents the canonical splice site (i.e., when the alternate allele is GT at the donor or AG at the acceptor site). *noncanonical_splice_flank*: variants in exons that are flanked by noncanonical splice sites. Some of these exons could be due to spurious exon annotations in the gene models. *Small_intron*: variants in introns <15 bp long.

### Pathogenicity prediction for pLoF mutations

To predict the pathogenicity of pLoF variants, we trained a random forest model to differentiate between benign, heterozygous, and homozygous disease-causing premature stop variants. For the training data, we only used premature stop variants caused by single-nucleotide polymorphisms because indel calling methods are not yet robust. The benign variant set includes homozygous variants from 1KG. Premature stop mutations leading to disease were obtained from HGMD. To minimize errors due to mistakes in HGMD, we only used high-confidence mutations labeled as “DM” (disease-causing mutations) in HGMD. We used the variation and gene-specific features that are output by ALoFT to build the classifier. We also included gene/transcript-specific features, which take into account the effects of length and the background mutation rate for each gene.

As training data, we identified benign premature stop variants as SNPs that are homozygous in at least one individual in 1KG. Premature stop mutations from HGMD are classified as those causing recessive or dominant disease based on ‘recessive’ and ‘dominant’ genes curated from the Online Mendelian Inheritance in Man database, OMIM^61, 62^. The training data consist of variants from autosomes only. Mutations that lead to dominant inheritance of diseases can do so both via loss-of-function mechanisms as well as gain-of-function mechanisms. However, it is reasonable to assume that most pLoF variants in dominant disease genes cause loss of function. Nonetheless, we only included dominant genes predicted to be haplo-insufficient^22^ in the training data to make sure that we are predominantly probing loss-of-function effects. The final training data set was derived from 397 benign premature stop variants (in 380 genes), 3300 dominant premature stop variants (in 136 genes), and 5342 recessive premature stop mutations (in 796 genes) (Supplementary Table 2).

In order to classify loss-of-function mutations, descriptive features are transformed into binary values - −1” and “1”, e.g., whether or not truncating a PFAM domain. Missing values are replaced with the weighted average of the three prediction classes. We then use a random forest algorithm to train our model and evaluated the performance with 10-fold cross-validations. To reduce bias, we included only one variant per gene in the training data for the benign and recessive classes. The average number of dominant mutations per gene is 24 (Supplementary Table 2). Therefore, we randomly selected three variants per gene for the dominant class in order to obtain a reasonably balanced training data set. The variant is picked randomly from the list of mutations and the longest affected transcript is used. Thus, each training model was based on 380 benign premature stop variants, ~341 dominant mutations, and 796 recessive mutations. Stratified sampling is used in the random forest model to achieve balanced three-class training.

We repeated this process 40 times. We calculated multi-class AUC for the test set using the methodology developed by Hand and Till^69^. We assigned the class with the highest probability as the predicted outcome.

### Classifier performance evaluation

In cases where ALoFT returns a similar probability of classification between classes, there is uncertainty in the predicted class. By calculating the standard deviation of class probabilities across our 40 trained random forest models, we obtain a 95% confidence interval for ALoFT predictions. If the confidence interval of the predicted class probability overlaps with the confidence interval of either of the two less likely classifications (single-sided test), we attach the label ‘Low Confidence’ (*p* > 0.05) to the prediction. Otherwise the prediction is labeled ‘High Confidence’ (*p* < 0.05).

Supplementary Fig. 5 shows the precision calculations for 5 out of the 40 training models. Precision is calculated as the fraction of true positives among predictions. As an example, for recessive predictions, we counted the number of correct predictions as true positives, the rest of the recessive predictions as false positives.

$${\rm{Precision = }}\frac{{{\rm{True}}\,{\rm{positives}}}}{{{\rm{True}}\,{\rm{positives + False}}\,{\rm{positives}}}}$$

Recall is calculated as:

$${\rm{Recall = }}\frac{{{\rm{True}}\,{\rm{positives}}}}{{{\rm{True}}\,{\rm{positives + False}}\,{\rm{negatives}}}}$$

We evaluated the robustness of the classifier by using several different training data sets for the prediction. The classifier performs well for all the training data sets as shown in Supplementary Table 3.

Olfactory receptor genes have many pseudogenes and accumulate many LoF mutations^70^. Therefore, the training data for benign pLoF variants have a higher proportion of high-frequency pLoF variants from this class of genes. In order to avoid any potential bias arising due to this factor, we validated the robustness of our model by excluding olfactory receptors. Similarly, we show that the model performs well whether we choose variants from the longest isoform of a gene for the training data or choose any one of the isoforms of the gene. In addition to LoF effects, truncating mutations can also lead to gain of function. However, gain-of-function mutations are difficult to model systematically as the effect of a variant is very context dependent. In order to minimize errors that might arise due to inadequate modeling of gain-of-function effects and focus only on LoF, we use predicted haploinsufficient genes as the dominant training set in the final model. However, we show that even a model where the training data for the dominant class is derived from all dominant genes, the prediction is robust.

### Determining feature importance

In Supplementary Fig. 2, the importance of a feature is calculated by evaluating the decrease in mean accuracy of the test set when the value of the feature is randomly permuted. The importance plot is not directly interpretable because some of the prediction variables are correlated. The description of the features can be found in Supplementary Data 1.

To further evaluate the features important for the classification, we built several prediction models using different sets of features for the training. Supplementary Table 4 shows the features used for prediction and their corresponding multi-class AUC of the test set.

### Application of ALoFT to sequencing study data

We applied our method to classify Mendelian pathogenic mutations discovered in the Center For Mendelian Genomics studies (CMG)^33^. After excluding training variants, there are 3 dominant and 5 recessive premature stop mutations. We also obtained GERP and CADD^32^ scores for these variants (Fig. 2b).

ClinVar^31^ variants were obtained from https://github.com/macarthur-lab/clinvar. In order to validate ALoFT predictions, we first excluded all ClinVar variants in genes that were used in the training set. We then labeled the remaining ClinVar variants as those leading to disease via the dominant or recessive mode of inheritance using an orthogonal list of dominant/recessive genes obtained from Berg et al.^71^ To avoid potential bias that might arise due to enrichment of disease variants in particular genes, we randomly picked one variant per gene for the analysis shown in Fig. 2a. The final set used to validate ALoFT contains 197 variants in genes known to cause disease through the dominant mode of inheritance and 111 variants in recessive genes.

We collected de novo premature stop mutations from four autism studies^34–37^. There are 19 and 53 mutations in siblings and probands, respectively. Most individuals have one de novo premature stop mutation (Supplementary Table 5). The prediction results are included in Supplementary Data 2 (2 out of 53 proband mutations overlap our training data and are excluded in Fig. 3a).

We obtained the list of 33 confident autism genes (FDR < 0.1) from Rubeis et al.^39^ and observed that dominant disease-causing score for premature stop variants in these genes are significantly higher than those in other genes (Only de novo pLoFs in probands are used; *p*-value: 5e−3; Wilcoxon rank-sum test; Fig. 3b).

We obtained somatic premature stop mutations from Alexandrov et al.^40^. This includes 6535 exomes in 30 different cancer types. Cancer genes are from the COSMIC cancer gene consensus^72^.

We used ALoFT as a tool to distinguish passenger vs. driver mutations in tumors with high mutation burden. For this evaluation, we used ALoFT to identify deleterious LoF mutations. We calculated the ratio of deleterious LoF mutations to total pLoF mutations for the 6535 exome samples. We binned patient samples with at least one deleterious LoF mutation according to total mutational burden.

We applied our method to classify premature stop variants in the healthy cohort of 1092 individuals from the 1KG data. Among the 5495 premature stop variants (excluding chrX), 148, 3070, and 2277 variants are predicted as dominant, recessive, and tolerant, respectively (Supplementary Data 4).

### Estimating LoF mutation burdening

In order to estimate the burden of deleterious LoFs in an individual genome, we calculated the average number of premature stop variants predicted to be deleterious by ALoFT using data from 1KG Phase 1, 1KG Phase 3, and ExAC. Numerous confounding factors make it difficult to compare genetic variation data from resequencing studies. For example, the accuracy of variant calls varies depending on the sequencing depth, and different data sets use different variant calling algorithms and different metrics to evaluate the quality of variants resulting in differing sensitivity and specificity of variant calls. Also, whole-genome sequencing and whole-exome sequencing provide different genomic coverage, and among exome sequencing studies, different exome capture platforms may have different definitions of exome and different target enrichment efficiency.

1KG data consist of data obtained both based on exome capture as well as whole-genome sequencing, whereas ExAC is based on exome capture data.

To minimize errors arising from the above-mentioned factors, we used a filtering approach described below to obtain a conservative estimate of the burden of deleterious premature stop variants in an individual genome.

We used high-confidence variants for the calculation of per individual statistics for 1000 Genomes as described below. (1) While ALoFT provides several flags that identify likely false positive variant calls arising due to mismapping and annotation errors, we conservatively excluded only those pLoF variants that correspond to the ancestral allele as they are unlikely to result in loss of function. (2) Variants present at >12 alleles (~1% frequency for phase 1 and ~0.5% for phase 3) in either the European or African-American population of the 1KG cohort, but absent in the ESP6500 cohort were also removed as likely erroneous calls. (3) For the 1KG Phase 1 set, only variants called from exome sequencing (not available for Phase 3) were included in order to make a fair comparison with the ESP6500 data that is also based on exome capture. We calculated per individual statistics for predicted dominant, recessive, and benign premature stop mutations and is shown in Supplementary Table 6 and Supplementary Fig. 6. Per individual calculations are based on 246 individuals of African ancestry and 379 individuals of European ancestry for 1KG Phase 1; 661 individuals of African ancestry and 503 individuals of European ancestry for 1KG Phase 3. For ExAC per individual calculation, no filtering was applied as we do not want to remove true variant calls that might be present in this data set due to higher sequence coverage. Furthermore, ExAC contains data aggregated from several disease exome sequencing projects such as inflammatory bowel disease, GoT2D (Type 2 diabetes) consortium, myocardial infarction genetics consortium etc. and some of the variants might be true disease-causing variants. Thus, our approach provides a lower estimate of the number of potentially deleterious pLoF variants in healthy individuals based on the value from 1KG Phase1 calculation.

### Data availability

The ALoFT software can be downloaded from aloft.gersteinlab.org. All ancillary files needed to run the program are included with this download and described in the Methods section. All analyzed data have been included as Supplementary Data 1–5. Pre-calculated exome-wide ALoFT scores for all base substitutions that potentially lead to a premature stop codon can be downloaded for both HG19 and GRCh38 human genome reference from aloft.gersteinlab.org.

## Electronic supplementary material

Supplementary Information

Supplementary Data 1

Supplementary Data 2

Supplementary Data 3

Supplementary Data 4

Supplementary Data 5
