# Supplementary material for: Using ALoFT to determine the impact of putative loss-of-function variants in protein-coding genes
Source: Nat Commun. 2017 Aug 29;8:382. doi: 10.1038/s41467-017-00443-5 (PMC5575292; doi:10.1038/s41467-017-00443-5)
Supplement: Supplementary file 1 — Supplementary Information [file 41467_2017_443_MOESM1_ESM.pdf]

File Name: Supplementary Information
Description: Supplementary Figures and Supplementary Tables.

File Name: Supplementary Data 1
Description: Descriptions of features used to train the ALoFT model.

File Name: Supplementary Data 2
Description: ALoFT predictions for de-novo pLoF variants in four autism studies.

File Name: Supplementary Data 3
Description: Somatic mutation counts per gene from Alexandrov et al. (PMID: 23945592).

File Name: Supplementary Data 4
Description: ALoFT predictions for 1KG Phase 1 premature stop variants.

File Name: Supplementary Data 5
Description: ALoFT predictions for homozygous pLoF variants from Narasimhan et al. (PMID: 26940866).

**MISMAPPING**	Number of paralogs to genes containing pLoF<br>pLoF variant in segmental duplication<br>Number of pseudogenes of genes containing pLoF
**ANNOTATION ISSUES**	Alternative allele is ancestral allele<br>NAGNAG pattern indicating alternative splice sites<br>Alternative allele is the consensus splice site<br>pLoF containing exon flanked by non-canonical splice sites<br>Splice variants in short introns (<15bp)<br>pLoF within first or last 5% of coding sequence
**NETWORK**	Shortest path to disease-causing gene<br>Proximity parameter
**EVOLUTIONARY**	GERP score<br>GERP element<br>dN/dS (macaque)<br>dN/dS (mouse)<br>Percentage of conserved exons removed due to truncation. Calculated as the fraction of removed exons covered by GERP-constraint elements
**FUNCTIONAL INTERPRETATION**	NMD prediction<br>In PFAM, SMART domains<br>PFAM, SMART domain lost due to truncation<br>In trans-membrane, signal peptides<br>Transmembrane domain, signal peptides lost due to truncation<br>In SCOP domain, disordered region<br>SCOP domain, disordered region lost due to truncation<br>In post-translational modified sites (PTM)<br>PTM lost due to truncation
**OTHER**	1KG, ESP6500, ExAC allele frequency<br>Partial/full pLoF (pLoF affecting some isoforms of a gene/ all isoforms)<br>Coding variant annotations using VAT[1] tool

Supplementary Table 1: Annotations output by ALoFT for a putative loss-of-function (pLoF) variant

Dataset	Used as	# Genes	# Variants
Benign homozygous variants from 1KG	Training	380	397
Heterozygous1KG variants	Test	3,997	5,170
HGMD dominant	Training	136	3,300
HGMD recessive	Training	796	5,342
Other HGMD variants (excluded training variants)	Test	868	6,145

Supplementary Table 2: HGMD and 1KG premature stop variants used for training and testing

No.	Training datasets	Multi-class AUC
1	Dominant genes (includes only haplo-insufficient genes); Longest transcript	0.970
2	Same as 1, except removed all Olfactory receptor genes	0.969
3	Same as 1, except randomly picked one transcript	0.968
4	Same as 1, except used all dominant genes (without haplo-insufficiency filter)	0.934

Supplementary Table 3: Robustness of method with respect to training data

No.	Features	Multi-class AUC
1	ESP6500 and ExAC features (Allele frequency, presence/absence of allele)	0.787
2	Network features	0.807
3	Functional features	0.852
4	Evolutionary features	0.864
7	All features	0.970

Supplementary Table 4: Classifier performance evaluation

	Number of *de novo* premature stop mutations (Number of samples)
Siblings	1 (19)
Autism males	1 (33); 2 (2)
Autism females	1 (14); 2 (1)

Supplementary Table 5: Number of *de novo* premature stop mutations per individual

1KG Phase 1 (1,092 individuals)

Predictions	Number of premature stop variants	Number of premature stop alleles per individual Mean (Median)	
		Pre-filtering	After-filtering
Dominant	148	0.83 (1)	0.11 (0)
Recessive	3,070	7.67 (7)	2.77 (2)
Benign	2,277	126.1 (125)	50.2 (50)

1KG Phase 3 (2,504 individuals)

Predictions	Number of premature stop variants	Number of premature stop alleles per individual Mean (Median)	
		Pre-filtering	After-filtering
Dominant	307	1.02 (1)	0.15 (0)
Recessive	8,842	9.26(9)	4.55 (4)
Benign	3,829	104.2 (103)	65.7 (65)

ExAC (60,706 individuals)

Predictions	Number of premature stop variants	Number of premature stop alleles per individual Mean
Dominant	2,907	3.1
Recessive	88,539	16.7
Benign	12,060	76.3

Supplementary Table 6: Pathogenicity predictions of premature stop variants and average per individual statistics for 1KG Phase1, Phase 3 and ExAC cohort

For ExAC per-individual calculation, no variants were removed by filtering as we do not want to remove true variant calls that might be present in this dataset due to higher sequence coverage. Criteria for filtering of variants are described in the Methods.

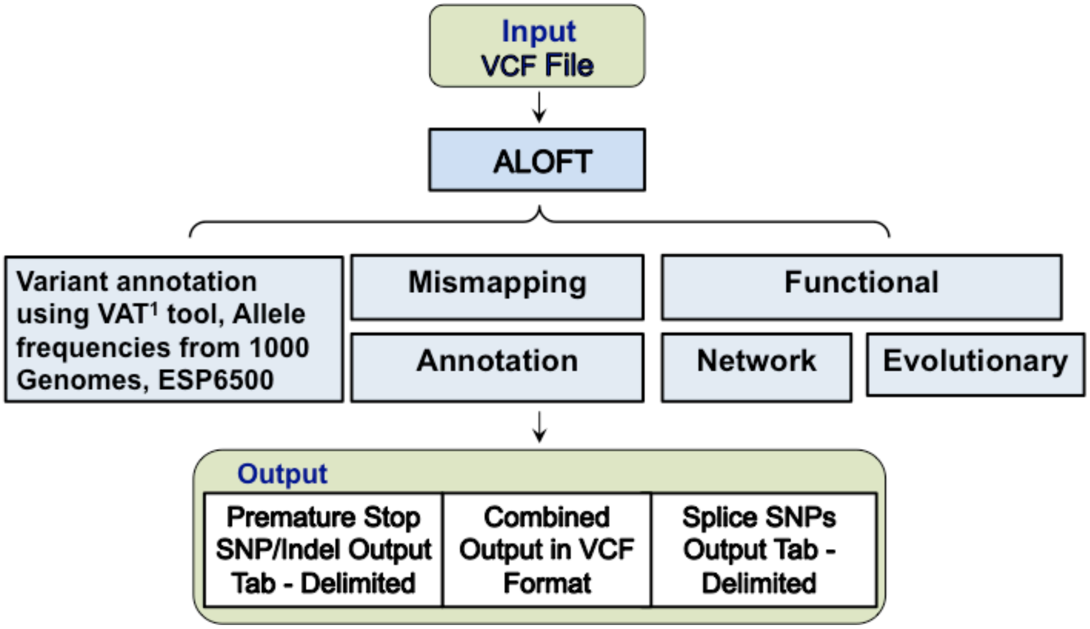

Supplementary Figure 1 – ALoFT annotation pipeline

Input data, intermediate processing steps, and output data are shown in this schematic representation of the ALoFT pipeline.

[1]Habegger, L. *et al.* VAT: a computational framework to functionally annotate variants in personal genomes within a cloud-computing environment. *Bioinformatics* **28,** 2267–2269 (2012)

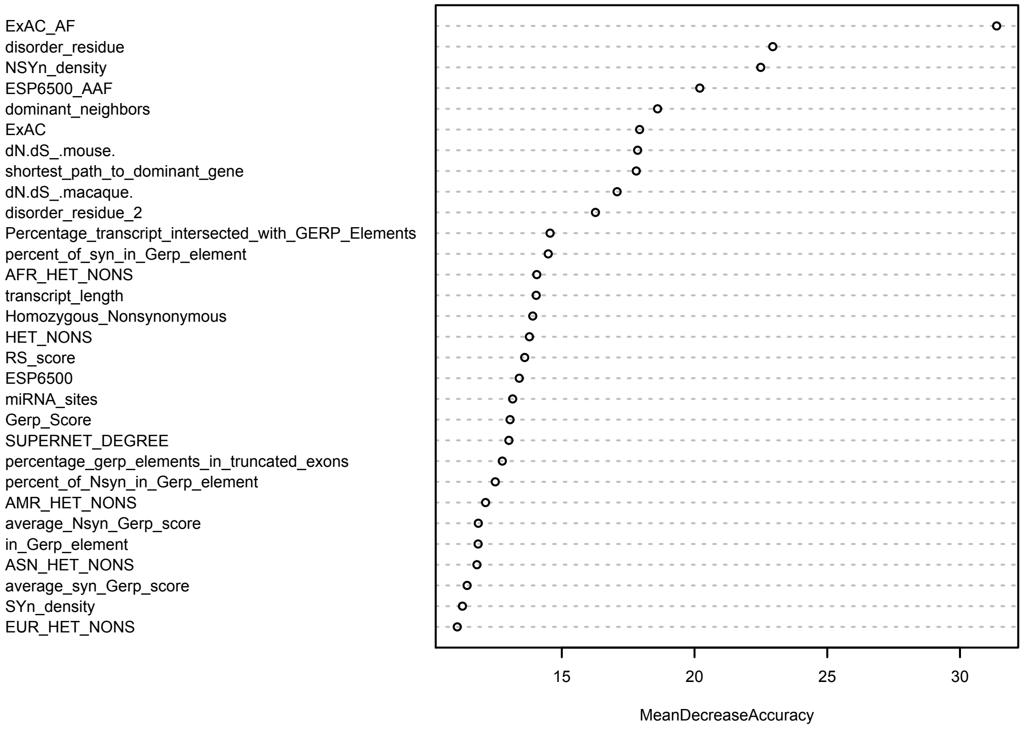

Supplementary Figure 2 – Importance plot

Features used to train the ALoFT model are ranked by relative importance. This importance measure is based on relative change in predictive accuracy of the ALoFT classifier upon removal of a given feature.

.

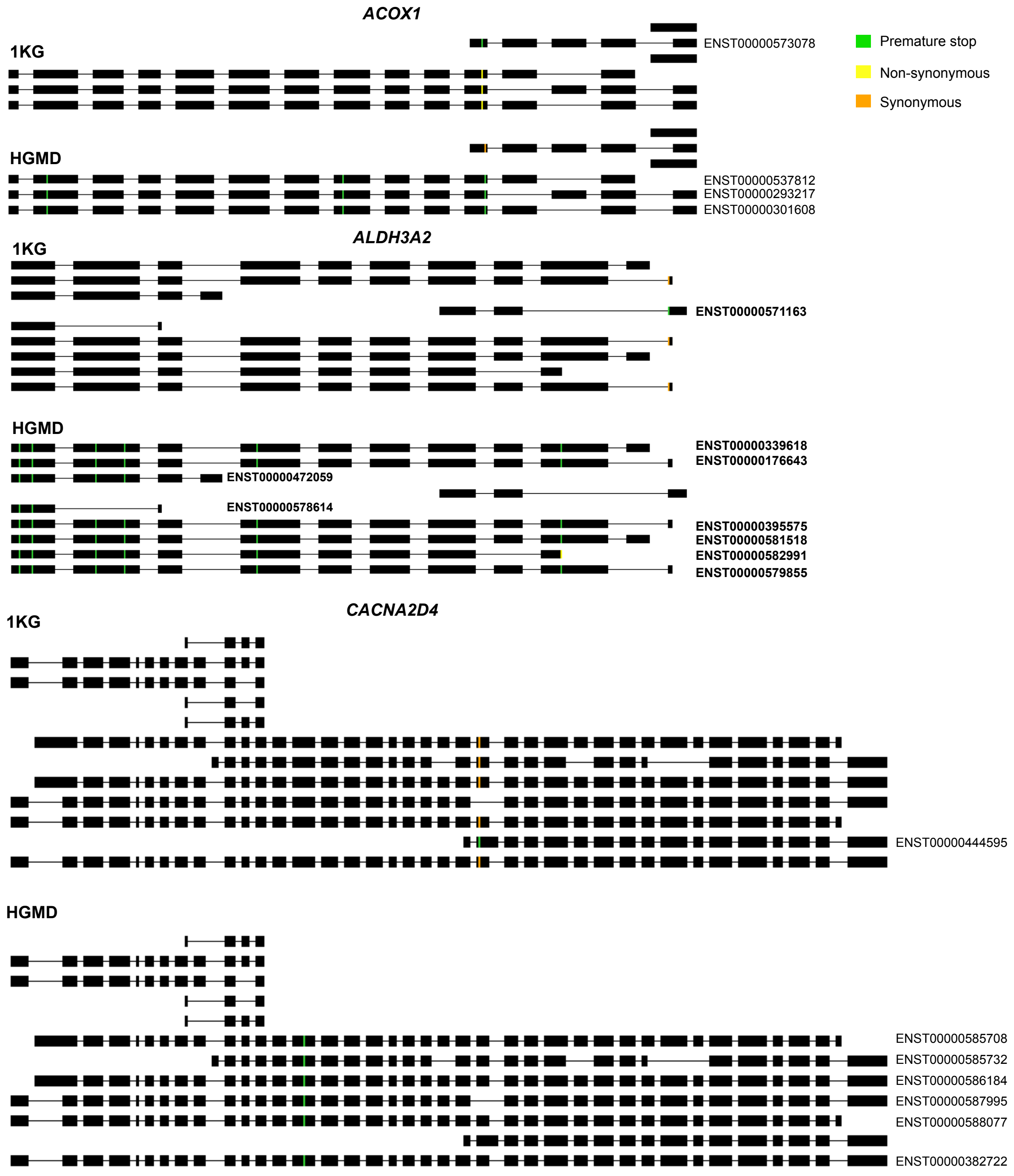

Supplementary Figure 3 – Examples of HGMD and 1KG pLoFs on different isoforms of a gene

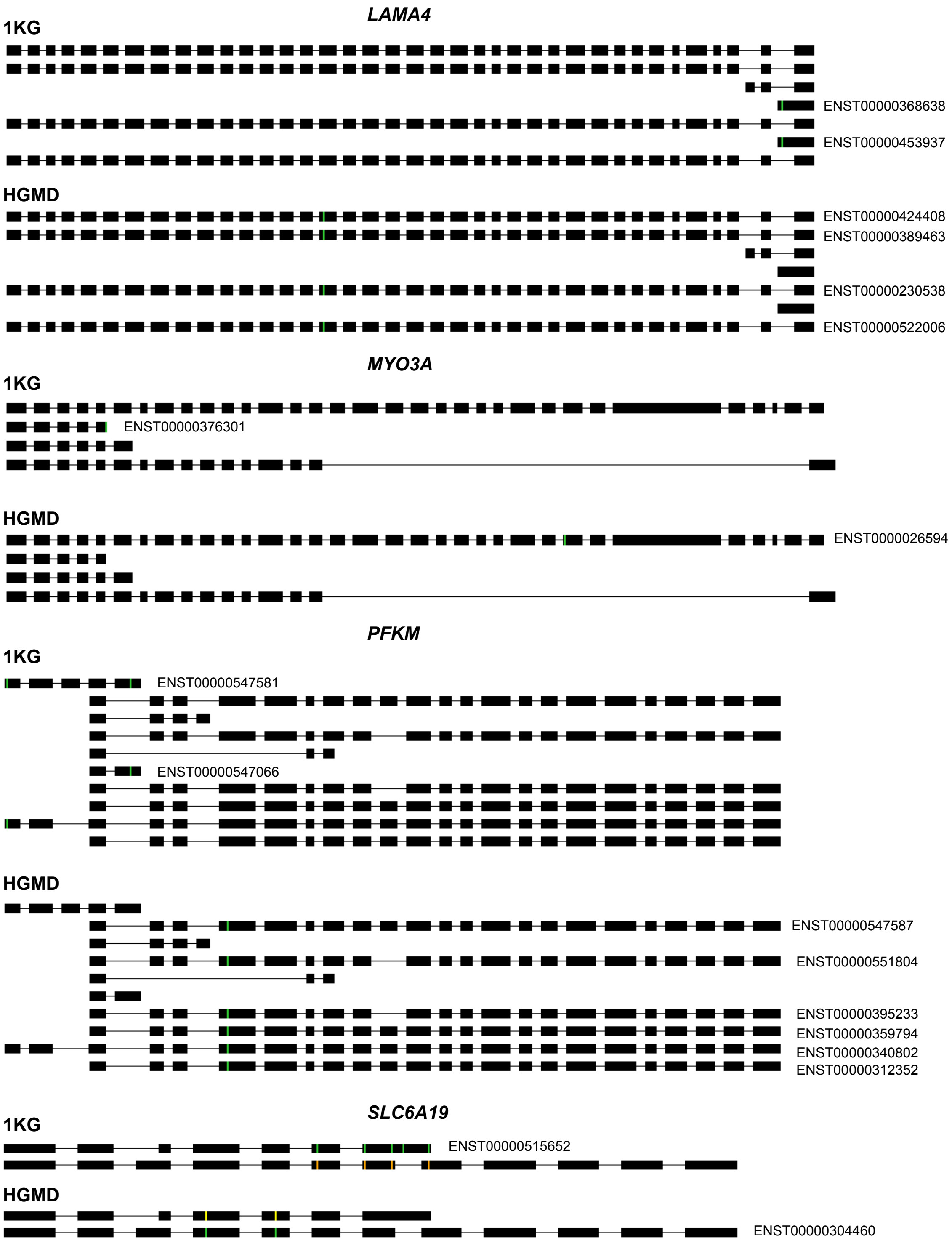

Supplementary Figure 3 – Examples of HGMD and 1KG pLoFs on different isoforms

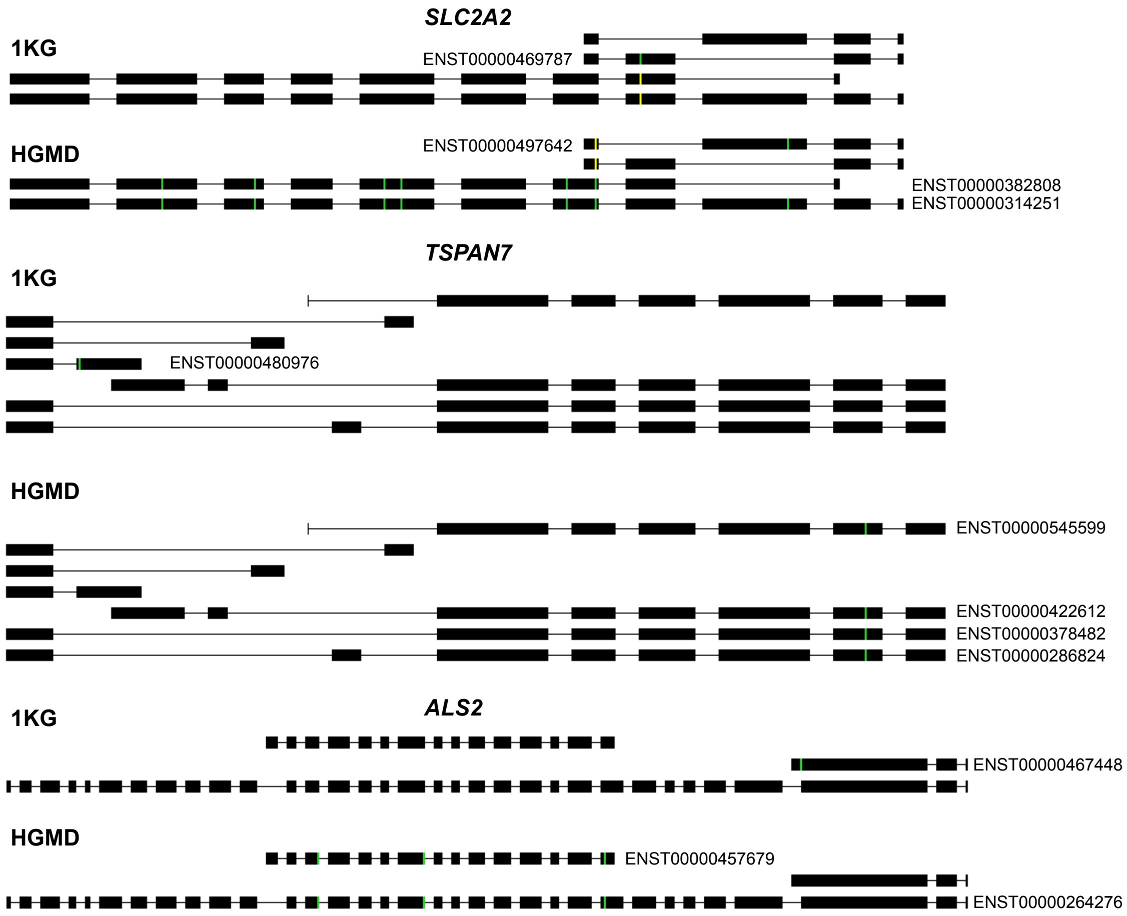

Supplementary Figure 3 – Examples of HGMD and 1KG pLoFs on different isoforms of a gene

Intronic and exonic regions of transcript isoforms for the genes *ACOX1*, *ALDH3A2*, *CACNA2D4, LAMA4, MYO3A, PFKM, SLC6A19, SLC2A2, TSPAN7, and ALS2* are depicted and identified by ENSEMBL transcript ID. Genetic variants from the Human Gene Mutation Database and 1KG cohort affecting these genes are marked in the locations where they occur. These variants are colored according to variant type. Premature stop variants, non-synonymous variants, and synonymous variants are distinguished.

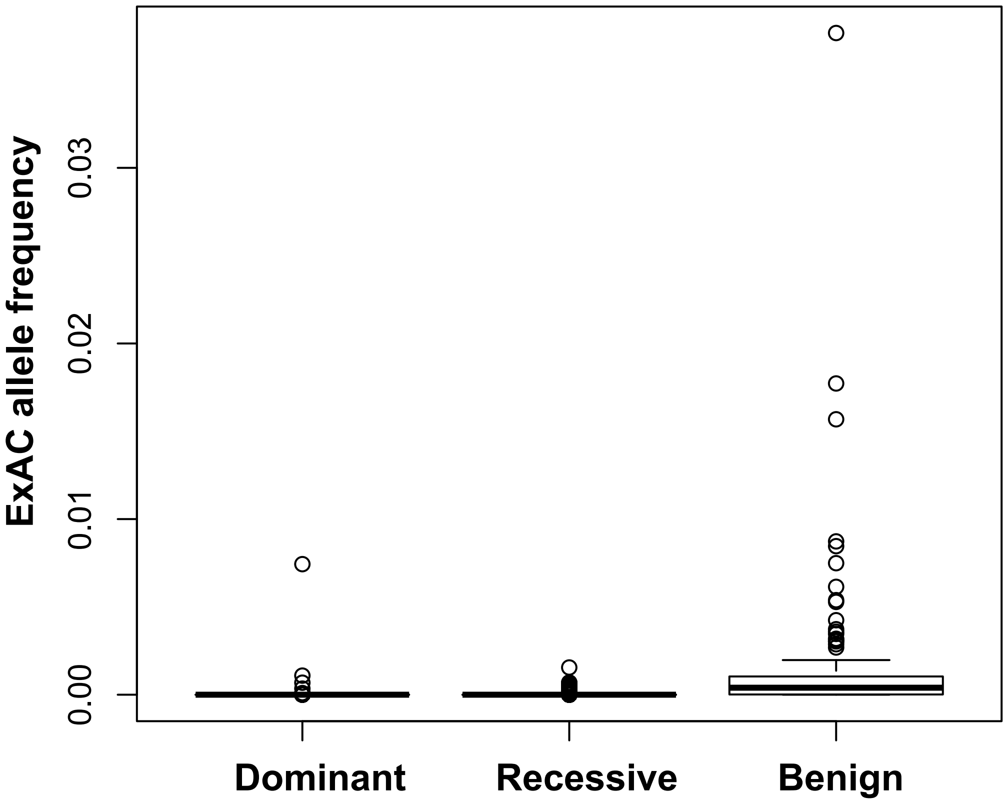

Supplementary Figure 4 – ExAC allele frequency distribution of HGMD premature stop variants in the dominant, recessive and benign classes

The global allele frequency of premature stop variants from HGMD in the ExAC database[33]. The median allele frequency values for mutations in dominant, recessive and benign classes are 0.0, 0.0 and 0.0004 respectively.

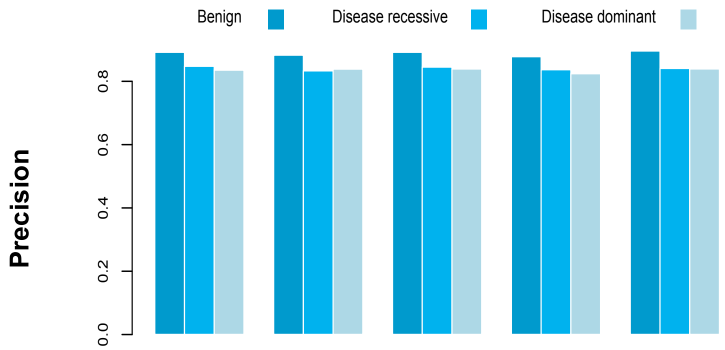

Supplementary Figure 5 – Precision plot for 5 models

Of the 40 random forest models used to train the ALoFT, the precision of 5 of these models is shown for variants in each of the three classification categories (benign, recessive disease-causing, dominant disease-causing) with respect to training data.

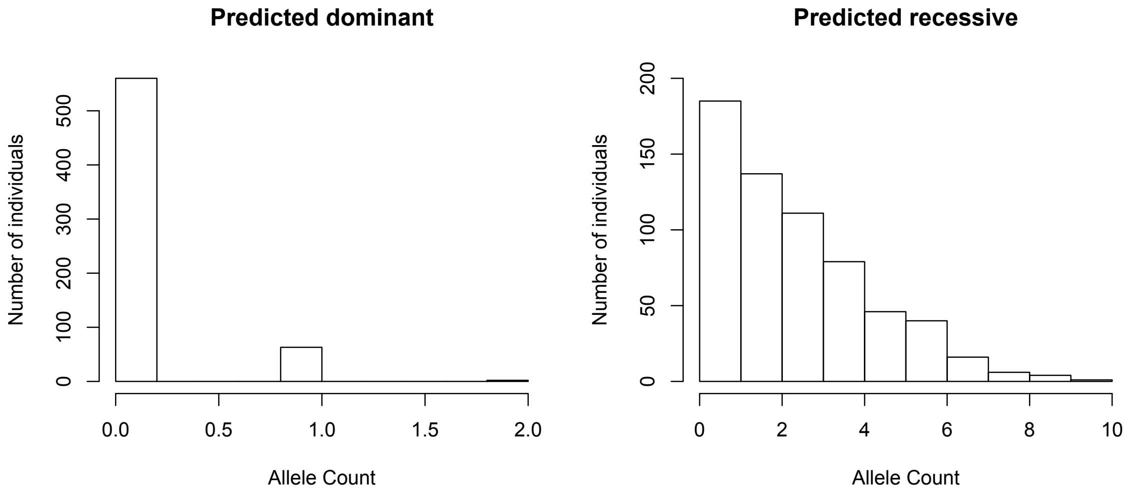

Supplementary Figure 6 – Distribution of predicted dominant and recessive premature stop alleles in the 1KG Phase 1 individuals

The total number of 1KG individuals with a given total count of either dominant (left) or recessive (right) classified premature stop variants.
